# Serological and Molecular Prevalence and Associated Risk Factors in Caprine Brucellosis, Northeastern Thailand

**DOI:** 10.1155/2024/9966352

**Published:** 2024-10-23

**Authors:** Sarinya Rerkyusuke, Sawarin Lerk-u-suke, Peerapol Sukon, Patchara Phuektes

**Affiliations:** ^1^Division of Livestock Medicine, Faculty of Veterinary Medicine, Khon Kaen University, Khon Kaen 40002, Thailand; ^2^KKU Research Program, Khon Kaen University, Khon Kaen 40002, Thailand; ^3^Department of Geographic Information Science, School of Information and Communication Technology, University of Phayao, Phayao 56000, Thailand; ^4^Research Unit of Spatial Innovation Development, School of Information and Communication Technology, University of Phayao, Phayao 56000, Thailand; ^5^Division of Anatomy, Faculty of Veterinary Medicine, Khon Kaen University, Khon Kaen 40002, Thailand; ^6^Division of Pathobiology, Faculty of Veterinary Medicine, Khon Kaen University, Khon Kaen 40002, Thailand

**Keywords:** brucellosis, meat goat, northeastern, risk factors, Thailand

## Abstract

Brucellosis is a significant zoonotic disease with global implications for animal and human public health. This study investigated the prevalence of caprine brucellosis in 39 meat goat herds in northeastern Thailand using serological and molecular methods. Seroprevalence, determined by the modified Rose Bengal test (mRBT), was negative, indicating no detectable antibodies against *Brucella*. However, real-time PCR identified *Brucella* spp. DNA in 11 samples from 8 herds. Intraherd prevalence varied from 0.0% to 9.09%, averaging 6.73% (95% CI, 4.74–8.72). Univariate analysis revealed significant risk factors associated with brucellosis at the herd level. Larger herd size correlated with increased brucellosis odds ratio (OR: 6.30; 95% CI: 1.07–36.93; *p*=0.041). Herds with multiple reproductive failures, including abortion, repeat breeding, and sterile, together with weak offspring, showed higher prevalence (OR: 9.37; 95% CI: 1.17–74.84; *p*=0.034). Multivariable analysis identified herd sizes over thirteen as a significant risk factor (OR: 10.20; 95% CI: 1.06–97.40; *p*=0.044). Notably, herds where owners were aware of direct transmission risks exhibited lower infection rates (OR: 0.05; 95% CI: 0.006–0.54; *p*=0.012). This study underscores the complementary role of molecular techniques alongside serological tests in detecting *Brucella* infection accurately. The findings highlight the importance of effective herd management, reproductive health monitoring, and owner education in mitigating brucellosis transmission. Implementing robust control measures, including stringent biosecurity protocols and enhanced stakeholder awareness, is crucial for controlling brucellosis in meat goat populations.

## 1. Introduction

Brucellosis is a highly contagious zoonotic disease transmitted from animals to humans, caused by bacteria of the genus *Brucella*, including *Brucella abortus*, *Brucella melitensis*, and *Brucella suis*. This disease affects various livestock species, including cattle, sheep, goats, pigs, camelids, and cervids [1]. While several countries, such as those in Western and Northern Europe, Canada, Japan, Australia, and New Zealand, are classified as brucellosis-free [2], the disease remains endemic in regions characterized by extensive livestock farming, including the Mediterranean basin, parts of Africa, Latin America, the Middle East, and Asia [2]. In Southeast Asia, brucellosis has been reported or suspected in countries such as Indonesia, Malaysia, Myanmar, Timor-Leste, and Thailand [3].

The persistence of brucellosis in these regions poses substantial health and economic challenges to the livestock sector. Clinically, the disease manifests primarily as reproductive failures, including abortions, stillbirths, and infertility, with additional impacts such as reduced milk production, weight loss, chronic infection, and, in severe cases, death [4, 5]. These consequences lead to diminished livestock productivity, primarily due to decreased reproductive efficiency and reduced meat and milk production, resulting in significant financial losses for farmers. Furthermore, asymptomatic cases in nonpregnant animals [6] complicate detection and control efforts. The financial burden extends beyond veterinary treatment and diagnostic testing, as necessary control measures, such as culling, further exacerbate the economic strain. Moreover, brucellosis-related trade restrictions limit the export of livestock and animal products, reducing potential revenue from international markets.

Understanding the epidemiology and risk factors associated with brucellosis is crucial for effective management and control. The primary modes of transmission include direct contact with infected animals or exposure to contaminated environments [6–8]. *Brucella* bacteria are present in reproductive fluids, placentas, fetal tissues, and vaginal discharges, making parturition a high-risk period for transmission [6, 9]. Ingestion of contaminated feed, water, or milk also poses a significant risk, as bacteria may be excreted in the milk of infected animals [10, 11]. Venereal transmission, along with nonvenereal transmission through behaviors such as sniffing or licking the external genitalia, further contributes to intraherd spread [6]. The bacteria can survive for extended periods in the environment, particularly in moist and shaded areas, facilitating indirect transmission via contact with contaminated surfaces, including bedding, feed, water troughs, and farm equipment [6]. Although less common, airborne transmission through the inhalation of particles from contaminated feces or urine has also been reported [9].

Previous studies have identified several risk factors associated with brucellosis seropositivity in goats in northern and central Thailand. Key factors include herd size, reproductive issues, participation in brucellosis testing programs, the source of new animals, farm disinfection practices, communal grazing, and a history of clinical signs of brucellosis [12, 13]. In northeastern Thailand, there is clinical evidence linking reproductive disorders to bacterial infections, with particular concern over the communal use of bucks within herds [14]. Nevertheless, further research is necessary in this region to examine factors such as herd structure, management practices, reproductive history, and awareness of transmission pathways and clinical signs of brucellosis in both livestock and humans.

Human brucellosis outbreaks, primarily due to zoonotic transmission, have been documented in northern, southern, western, and central Thailand [15–20]. The disease is characterized by chronic symptoms, including recurring fever and severe complications, particularly in pregnant women, imposing a significant burden on healthcare systems and hinders the socioeconomic progress of affected individuals [21]. This highlights the critical importance of understanding the epidemiology of brucellosis as a zoonotic disease. Given the significance of goat farming in northeastern Thailand, investigating the prevalence and transmission dynamics of *Brucella* spp. within this region is imperative. However, there is a notable gap in the literature regarding the prevalence of brucellosis and its associated risk factors in this area. This study aims to address this gap by determining the prevalence of *Brucella* spp. and identifying the associated risk factors in meat goat herds. The findings will be instrumental in developing effective strategies for the control and prevention of brucellosis in northeastern Thailand.

## 2. Materials and Methods

### 2.1. Ethical Approval

Sample collection from animals in this study was approved by the Institutional Animal Care and Use Committee (IACUC) under record number IACUC-KKU-64/66, granted on June 19, 2023. Additionally, approval for data collection from owners was obtained from the Center for Ethics in Human Research, with record number HE672063, on April 3, 2024. These approvals indicate compliance with the ethical standards and regulations governing research involving both animal and human subjects.

To ensure confidentiality, all data were anonymized and aggregated at the village level, with no personal identifiers disclosed. The maps presented in this paper were designed to protect respondents' privacy and did not include specific addresses.

### 2.2. Study Area and Design

A cross-sectional study was conducted from August 2023 to May 2024, examining a total of 39 meat goat herds located in Chaiyaphum and Khon Kaen provinces, which are known for their high densities of meat goat farming in Northeast Thailand [22].

• In Chaiyaphum Province, investigations were carried out across 3 districts: Ban Thaen (4 herds), Kaset Sombun (2 herds), and Noen Sa-nga (2 herds).
• In Khon Kaen Province, the study encompassed 10 districts: Ban Haet (2 herds), Chum Phae (2 herds), Khao Suan Kwang (2 herds), Mancha Khiri (2 herds), Muang Khon Kaen (7 herds), Nam Phong (3 herds), Nong Ruea (2 herds), Phra Yuen (1 herd), Phu Wiang (4 herds), and Si Chomphu (6 herds).

### 2.3. Animal and Sampling Procedures

The estimated population of meat goats in Chaiyaphum and Khon Kaen provinces is approximately 64,936 goats [22]. These goats are raised in smallholder herds that operate as communal entities. Serum and vaginal swab samples were collected for serological and molecular testing, respectively.

Serum samples were collected from goats older than 6 months, in accordance with the criteria outlined in the brucellosis surveillance program [23, 24]. Since each herd under investigation consisted of fewer than 49 mature animals, serum samples were collected from all goats on each farm, adhering to the guidelines of the Brucellosis surveillance program [23].

Determination of sample size for vaginal swab samples for molecular detection considered an expected frequency of 50%, a margin of error of 5%, a design effect of 1.0, and a single cluster due to unknown prevalence of *Brucella* spp. infection in these populations. This resulted in a calculated sample size requirement of 382 samples, as computed using EPI INFO™ for Windows Version 7.2.5.0.

We employed a combined approach using both convenience and random sampling methods to ensure a robust and representative analysis. First, random sampling was utilized to select the study area, eliminating selection bias and ensuring that the chosen area reflects broader conditions. Within this selected study area, we then applied convenience sampling to target farmers who voluntarily agreed to participate in the research. In total, samples were collected from 39 herds across the study area, comprising 515 serum samples and 327 vaginal swab samples from 515 goats. Among these, both serum and vaginal swab samples were obtained from 327 does within the first three months postpartum or during a nonpregnant status. Serum samples were exclusively collected from 40 bucks and 148 pregnant goats. None of the meat goat herds had a history of brucellosis vaccination.

For serum sample collection, blood was obtained from mature goats aged over 6 months through jugular puncture, ensuring the collection of approximately 5 mL of whole blood under aseptic conditions in Vacutainer red tubes. A total of 515 blood samples were collected from the targeted goat population and promptly transported to the laboratory at the Faculty of Veterinary Medicine, Khon Kaen University, within 6 h to preserve sample integrity. Upon arrival, the samples underwent centrifugation to separate the serum from cellular components, and the resulting serum was stored at −20°C until further analysis.

Vaginal swab samples were collected from 327 does using Puritan 6″ sterile Rayon Tipped Applicator dry swabs under strict aseptic conditions. Swabs were immersed in 1 mL of phosphate-buffered saline (PBS) solution (0.01 M, pH 7.4) in sterile conical plastic tubes and promptly transported on ice to the designated laboratory within 6 h. In the laboratory, rigorous biosafety protocols consistent with biosafety level 2+ standards were strictly adhered to during sample processing. Swabs underwent thorough mixing using a vortex mixer for 15 s to ensure homogeneity. Samples containing sediment were subjected to brief centrifugation at 3000 rpm for 5 min to facilitate sedimentation, allowing for the collection of approximately 1 mL of the supernatant. The collected supernatant was extracted for DNA analysis.

### 2.4. Serological Test

Serological testing for brucellosis adhered to the WOAH standard procedure, beginning with the modified Rose Bengal test (mRBT) [25]. Positive samples identified during initial screening were subsequently confirmed using the complement fixation test (CFT) [25]. Both mRBT and CFT were conducted in an ISO/IEC 17025:2017 accredited laboratory by the Bureau of Laboratory Quality Standard, Department of Medical Science, Ministry of Public Health, Thailand.

The mRBT employed *Brucella abortus* antigen suspended in buffered diluents and stained with Rose Bengal dye, sourced from the Bureau of Veterinary Biologics, Department of Livestock Development, Thailand. Briefly, 25 *μ*L of *Brucella* antigen was mixed with 75 *μ*L of serum on a glass plate and spread uniformly over circles approximately 2 cm in diameter. The plate was rotated manually for 4 min according to WOAH standards [24]. Positive results were indicated by any degree of agglutination or clumping, while the absence of such reactions was considered negative. The mRBT demonstrated a sensitivity of 98.97%–100% and specificity of 96.4%–98.49% [26].

The CFT was used to detect antibodies against *Brucella* antigens. The veronal buffer ingredients for the CFT were obtained from ID Vet in France. The test followed the procedure outlined in the WOAH guidelines [25]. The undiluted test sera were inactivated in a water bath at 60°C for 30 min. In each row of the 96-well plates, 25 *μ*L of inactivated and 1/5 diluted test sera were dispensed. The antigen, diluted to a working strength of 1/10, was added to wells in rows B, D, F, and H, while veronal buffer was dispensed in the anticomplementary rows (A, C, E, and G). The antigen-test sera mixture and all control wells were incubated at 37°C for 30 min. Next, 25 *μ*L of working complement of verified strength was added to each well and incubated at 37°C for another 30 min. The control wells were handled in a separate plate. After that, 25 *μ*L of 1:1000 diluted hemolytic serum in 1% sheep red blood cell (SRBC) was added to each well. The plates were reheated at 37°C for 30 min. Once incubation was complete, the plates were kept at 4°C for 2–3 h, allowing any unlysed cells to settle before reading the results. Percent lysis was evaluated against the anticomplementary wells, with greater than or equal to 50% lysis considered positive and less than 50% lysis considered negative results [25]. The sensitivity and specificity of the CFT were reported to be 98.3% and 100%, respectively [26].

### 2.5. DNA Extraction

DNA extraction from vaginal swab samples was carried out using the DNeasy Blood and Tissue Kit (Qiagen) according to the manufacturer's instructions. Initially, 200 *μ*L of the sample was lysed with 20 *μ*L of proteinase K and 200 *μ*L of buffer AL. Subsequent purification steps included washing with 500 *μ*L of buffer AW1 followed by 500 *μ*L of buffer AW2 to eliminate impurities. The purified DNA was then eluted in buffer AE and stored at −20°C for subsequent PCR analysis.

### 2.6. Real-Time PCR Amplification

To detect *Brucella* spp. DNA in vaginal swab samples, a real-time PCR assay targeting the *bcsp31* gene was employed, following the methodology described by Probert et al. [27], with minor adaptations. The *bcsp31* gene is highly conserved among *Brucella* spp. and has been used as a molecular marker for the identification of *Brucella* species [27, 28]. Each PCR mixture consisted of 0.4 *μ*M of forward primer (5′-GCTCGGTTGCCAATATCAATGC-3′), 0.4 *μ*M of reverse primer (5′-GGGTAAAGCGTCGCCAGAAG-3′), 0.2 *μ*M of TaqMan probe (FAM-AAATCTTCCACCTTGCCCTTGCCATCA-BHQ-1), 10 *μ*L of 2x QuantiNova Probe PCR Master Mix (Qiagen), 3 *μ*L of DNA template, and DNase-RNase free water to reach a final volume of 20 *μ*L. PCR amplification and fluorescence detection were conducted using the CFX96 Touch Real-Time PCR Detection System (Bio-Rad Laboratories). The PCR protocol began with an initial denaturation step at 95°C for 2 min, followed by 45 cycles of denaturation at 95°C for 5 s and annealing at 58°C for 30 s. Positive controls containing plasmid DNA with the targeted *bcsp31* gene sequence of *Brucella abortus* and negative controls lacking template DNA were included in each PCR run to ensure accuracy and reliability. The sensitivity of the PCR was evaluated using diluted plasmid DNA at copy numbers 10^9^, 10^7^, 10^5^, 10^3^, and 10. PCR products from positive samples underwent gel purification using the MinElute Gel Extraction Kit (Qiagen) followed by DNA sequencing. The resulting nucleotide sequences were verified as *Brucella* spp. using the BLAST tool.

### 2.7. Questionnaires

Thirty-nine herd owners participated in structured interviews aimed at evaluating heard health status, management practices, and identifying potential risk factors associated with brucellosis in the studied populations. The questionnaire comprehensively covered aspects of herd management, including herd structure, herd and health management practices, and reproductive history. Specifically, the questionnaire assessed herd structure by inquiring about herd establishment and herd size. For herd and health management practices, the questionnaire evaluated aspects such as pasture management, the presence of other ruminant livestock or pests, adherence to good farm management practices, annual brucellosis testing, quarantine and movement control, the utilization history of bucks, mother-rearing systems, and parturition management, which included the provision of a separate parturition area and the use of gloves and masks during birthing assistance. Additionally, the questionnaire collected information on the incidence of reproductive failures, including abortion, repeat breeding, sterility, orchitis, and weak offspring (supporting 1).

The interviews also investigated farmers' knowledge regarding brucellosis transmission, self-reported symptoms possibly indicative of brucellosis. Detailed records of animal characteristics and clinical signs, encompassing gender, age, body condition score (BCS), and reproductive history, were meticulously recorded.

### 2.8. Data Analysis

To identify the risk factors associated with brucellosis, data obtained from questionnaires were analyzed using univariate analysis and multivariable logistic regression. Initially, univariate analysis was employed to identify variables associated with brucellosis positivity in both herds and individual animals, with a statistical significance threshold set at 0.05. OR and their corresponding 95% confidence intervals were calculated to evaluate the strength of these associations. Variables with a significance level of *p* < 0.1 in the univariate analysis were included in a multivariable logistic regression model, constructed using a backward stepwise method (entry criterion: *p* < 0.05; removal criterion: *p* < 0.10) to derive the final model. This approach aimed to identify the model with the lowest −2 Log likelihood, ensuring robust predictions of brucellosis outcomes. OR with 95% confidence intervals were calculated for the final model to assess the magnitude and direction of associations. All statistical analyses were performed using IBM SPSS Statistics Version 28.0 and MedCalc Version 22.021.

Additionally, Geographic Information System (GIS) was used to conduct the epidemiological analysis with QGIS, an open-source software platform (Version 3.36.0). The utilization of GIS represents a significant advancement in spatial epidemiology, enabling spatial mapping and visualization of brucellosis distribution and its associated risk factors in the study area.

## 3. Results and Discussion

### 3.1. Prevalence of Brucellosis in Meat Goat Herds

Results of real-time PCR, including nucleotide sequences, chromatograms, and BLAST search results, are presented in supporting Figures 1–4. The sensitivity of the real-time PCR was determined to be as low as 10 copy number of DNA while the specificity of positive PCR samples was confirmed through the analysis of the nucleotide sequences of the PCR products. The serological investigation revealed a negative seroprevalence for brucellosis, as assessed through the mRBT for screening antibodies. However, *Brucella* spp. DNA was detected in 11 samples from 8 herds using real-time PCR, resulting in a molecular prevalence ranging from 0.0% to 20.0% per herd (average 10.61%; 95% CI, 6.67–14.55). Intraherd prevalence, depicting brucellosis proportions within herds, ranged from 0.0% to 9.09%, averaging 6.73% (95% CI, 4.74–8.72) (shown in Table 1). Interestingly, all eleven PCR-positive samples were seronegative according to the mRBT technique. These animals were over 1 year old, had recently kidded, and were nonpregnant at the time of sampling. Reproductive histories in 2023 indicated normal kidding (*n* = 5), repeat breeding (*n* = 2), pseudopregnancy (*n* = 1), abortion (*n* = 1), and unknown histories (*n* = 2).

Detection of *Brucella* spp. DNA in 3.36% of the 327 samples using the *bcsp31* gene suggests exposure to a small number of organisms, potentially resulting in self-limiting or immunizing infections [29]. Animals may transition to a seronegative state as pathogen levels decrease [30], particularly within 14 days of infection when detectable antibody levels may be insufficient [31]. Nevertheless, such animals can remain infectious. The absence of detectable antibodies in infected cases may be attributed to low pathogen counts insufficient to elicit an immune response [30], highlighting the necessity of molecular methods for pathogen detection. The high sensitivity of real-time PCR, capable of detecting as little as 23 fg (corresponding about 7 genome copy equivalent) of *Brucella* DNA per reaction with 95% certainty in the previous study [32] and as few as 10 copies in our study, underscores its diagnostic utility. Identification of pathogen DNA through molecular methods is essential in cases of seronegative animals, as they can act as asymptomatic carriers and shedders, posing a permanent risk of disease transmission to other animals and humans [32]. Per WOAH guidelines, such animals should be removed from herds despite the typically chronic nature of brucellosis. Nonetheless, serological tests remain cost-effective and efficient for screening antibodies against *Brucella* infection in herds or flocks.

Our findings indicate a varied distribution of brucellosis within the studied populations. Discrepancies between seroprevalence and molecular prevalence may reflect different infection stages or diagnostic method sensitivities [30–32]. The observed prevalence rates emphasize brucellosis's significance as a potential threat to both animal and human health, underscoring the importance of effective disease control measures. Understanding the epidemiology and prevalence patterns within specific populations is crucial for implementing targeted control strategies, such as biosecurity measures, to mitigate disease spread. Additionally, this investigation recommends further sampling of vaginal swabs for molecular detection of *Brucella* in seronegative herds experiencing reproductive failures such as abortion, repeat breeding, or weak offspring.

## 4. Spatial Distribution of Brucellosis in Meat Goat Herds

**Figure 1:**
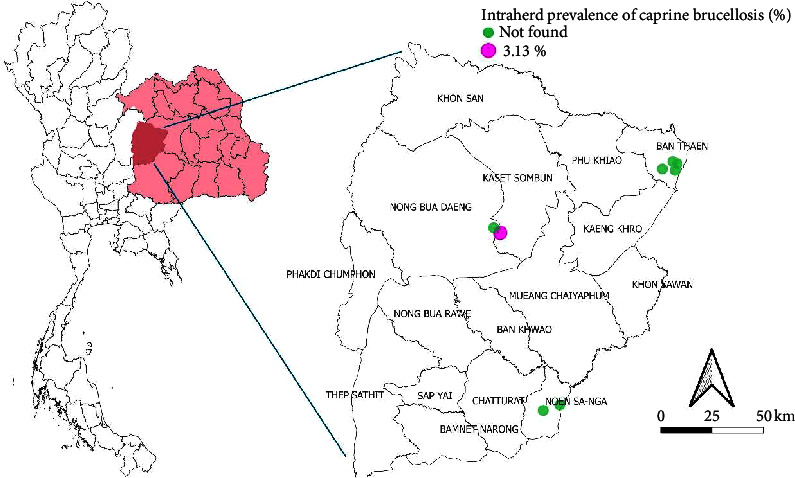
The spatial distribution of brucellosis in meat goat herds in Chaiyaphum province, Northeast Thailand.

**Figure 2:**
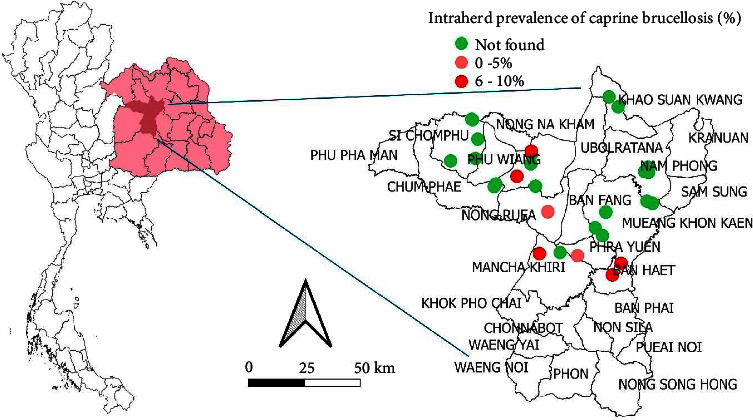
The spatial distribution of brucellosis in meat goat herds in Khon Kaen province, Northeast Thailand.

Figures 1 and 2 present the spatial distribution of brucellosis in meat goat herds across Chaiyaphum and Khon Kaen provinces, respectively. These visual representations depict the prevalence of brucellosis at both provincial and district levels. The findings from this study reveal heterogeneous prevalence rates across the study area, underscoring spatial variability in brucellosis distribution. The molecular prevalence of brucellosis was notably higher than seroprevalence, suggesting the shedding of *Brucella* spp. through vaginal secretions, even in clinically healthy goats [33–35]. The spatial epidemiology suggests that positive cases of brucellosis in the investigated herds may be linked to the communal sharing of bucks between herds, as well as the introduction of new animals from the same province or district. These practices likely contribute to the spread of brucellosis within the herds [21].

### 4.1. Risk Factors Associated With Brucellosis in Meat Goat Herds

According to our investigation, questionnaire data revealed that 87.2% (34/39) of herds experienced reproductive failures such as abortion (22/39), weak offspring (22/39), sterility (11/39), repeat breeding (5/39), and orchitis (4/39). Only 5 herds did not report these issues. The analysis explored correlations between brucellosis, identified through positive PCR assays, and various independent variables at both herd and individual animal levels. These variables included herd establishment duration, size, pasture management, reproductive history, buck circulation patterns, parturition management practices, quarantine measures, presence of vectors, manure handling, clinical signs resembling brucellosis symptoms, and knowledge of brucellosis transmission.

At the herd level, univariate analysis identified several significant factors associated with brucellosis in meat goat herds (*p* < 0.05). Particularly noteworthy, larger herd size (OR: 6.30; 95% CI: 1.07–36.93; *p*=0.041) and herds experiencing multiple reproductive failures (OR: 9.37; 95% CI: 1.17–74.84; *p*=0.034), including abortion, repeat breeding, and weak offspring history, were significantly associated with higher brucellosis rates. Interestingly, herds where owners were aware of direct contact transmission exhibited a lower infection rate (OR: 0.05; 95% CI: 0.006–0.54; *p*=0.012). Comprehensive details of all risk factors associated with brucellosis in meat goat herds are presented in Table 2.

Multivariable analysis at the herd level identified herd size over thirteen as a significant risk factor for brucellosis (OR: 10.20; 95% CI: 1.06–97.40; *p*=0.044).  Table 3 provides a detailed overview of risk factors associated with brucellosis in meat goat herds.

Previous studies have reported varying results regarding herd size as a risk factor for caprine brucellosis. For instance, studies in Nepal, Niger, and Ethiopia found that larger herd sizes were significant risk factors [36–39], while small size was not risk in Pakistan [40]. In Thailand, smaller herd sizes were identified as risks in some areas [19] but not in others [20]. The disparity in herd size as a risk factor may be region-specific.

Clinical reproductive disorders such as abortion, orchitis, repeat breeding, sterility, and weak offspring have been associated with seropositivity for brucellosis, *Coxiella burnetii*, and *Chlamydia* infections in previous studies [14]. However, in our study, multivariable analysis did not find these factors significantly associated with brucellosis in meat goat herds. Nevertheless, the history of reproductive failures may indicate infectious disease presence associated with reproductive failures, such as *Coxiella* infection in northeastern Thailand [41]. Previous studies have also linked abortion history in herds to increased seropositivity for brucellosis in Iraq, Ethiopia, and Tanzania [39, 42–45].

In a previous study conducted in Northeast Thailand, a seropositivity rate of 6.78% was reported for *Brucella* spp. in meat goat herds experiencing clinical reproductive disorders [14]. In our current study, PCR-positive samples were linked to reproductive failures such as repeat breeding (2 cases), pseudopregnancy (1 case), and abortion (1 case). Notably, a herd previously identified as seropositive for *Brucella* spp. [14] continued to experience abortion, despite culling a seropositive doe in 2019. Subsequent testing in 2023 showed seronegativity by mRBT but positivity by PCR, indicating ongoing circulation of *Brucella* spp. within the herd. To control brucellosis in affected herds, we recommend sampling vaginal, ocular, or nasal secretions for the molecular detection of *Brucella* spp. in both bucks and does, followed by culling of positive animals.

This study elucidates the intricate dynamics that influence disease prevalence within goat herds. The observed correlation between buck circulation and disease prevalence emphasizes the significance of animal movement in facilitating disease transmission, both within herds and potentially to humans [14, 46]. Implementing stringent biosecurity measures, including comprehensive health screenings for introduced animals and restrictions on interherd contact, is essential for mitigating the spread of disease [47]. Additionally, conducting annual brucellosis testing serves as a protective factor against the incidence of brucellosis on farms [14]. Furthermore, the impact of participants' knowledge regarding disease transmission routes underscores the importance of educational and awareness campaigns [47]. By equipping farmers with accurate information about disease risks and preventive measures, such initiatives can promote the adoption of best practices, ultimately reducing disease prevalence and enhancing overall herd health.

When conducting risk analyses, caution is warranted in interpreting nonsignificant associations. In this study, no significant correlation was found between the presence of other animal species (e.g., dogs, cats, and rodents) and brucellosis in positive herds. However, it is important to note that *Brucella* spp. can still be transmitted among ruminant species and companion animals [42, 44, 48–50]. Therefore, the presence of these animals should be a concern in goat herds, warranting continued attention and surveillance.

Furthermore, during parturition, farmers face a heightened risk of *Brucella* spp. exposure due to inadequate protective measures such as gloves (71.8%, 28/39) and masks (94.9%, 37/39) during assistance. Emphasizing the importance of personal protection, particularly glove use during birthing assistance, is crucial for minimizing transmission risks, as underscored in previous studies [39, 51].

Similarly, at the individual animal level, various potential risk factors for specific conditions provide valuable insights into their connection to *Brucella* spp. exposure and/or infection. While gender, age, and BCS did not show statistically significant associations in this study, they offer important clues about potential trends. This suggests that animals of all ages and genders were equally susceptible to infection or were exposed to a common source of infection, indicating the potential for disease emergence in the area. Similar findings of nonsignificant associations between gender and age have been reported in Nigeria and Ethiopia [40, 52–54]. The absence of a statistically significant difference in gender could be attributed to the smaller sample size of males and their shorter tenure in the herd, which may reduce exposure to the disease [54]. Age-related differences in brucellosis occurrence align with previous studies that identify brucellosis as primarily affecting sexually mature animals, though younger animals can acquire latent infections despite being more resistant to *Brucella* infection [55–57].

However, previous studies have indicated that gender, age, and BCS are consistent with high seropositivity for *Brucella* spp. infection, particularly in older or actively breeding animals [39, 42]. Further investigation is necessary to confirm these trends and gain a better understanding of their implications for disease management.

Our study has several limitations. We were not able to definitively confirm active infection with *Brucella* spp. and the related reproductive disorders in the examined herds due to constraints in our sampling methodology. We only collected vaginal secretions from apparently healthy does and did not obtain samples from aborted fetuses, placental tissue, or postpartum vaginal discharges. Moreover, our sampling was limited to mature goats aged over 6 months, which precluded the evaluation of infection in younger animals. Furthermore, further investigation is necessary to determine the exact species of *Brucella* implicated.

These findings underscore the interconnected dynamics of disease transmission within livestock populations. Factors such as gender, breed type, and clinical indicators not only influence individual susceptibility but also contribute to the broader epidemiology of infectious diseases like brucellosis within herds. Thus, implementing a comprehensive strategy encompassing individual risk factors and herd-level management practices is imperative for successful disease control and prevention.

In terms of individual factors associated with brucellosis, the univariate analysis indicated no significant difference in the likelihood of infection with brucellosis based on gender, age, and BCS (*p* > 0.05). A comprehensive overview of all individual risk factors associated with brucellosis is provided in Table 4. These findings suggest that gender, age, and BCS alone may not be strong predictors of susceptibility to brucellosis infection. Therefore, a holistic understanding of various risk factors is essential for developing effective prevention and control strategies against brucellosis in both animals and humans.

Seroprevalence studies have shown varying results regarding the impact of gender and age on brucellosis. For instance, some studies have reported no significant association between seroprevalence and sex or age, while others have found correlations between seroprevalence and factors such as abortion rate and parity number [58, 59]. Additionally, previous studies have identified older age (over 2 years) and female does as having higher risk factors for brucellosis [60]. Conversely, another study highlighted younger animals as significant risk factors, with no mention of gender or BCS as contributing factors [61].

Other potential risk factors for caprine brucellosis may stem from the production system, such as agropastoral or pastoral practices, as reported in previous studies [39]. However, in this observational study, the majority of the meat goat production (38 out of 39 herds) was sedentary, with all animals residing in private areas. This sedentary lifestyle may act as a confounding factor in the prevalence of brucellosis in this region. Further research is needed to explore the various factors influencing brucellosis transmission and to evaluate the effectiveness of control interventions aimed at reducing disease prevalence within affected populations. Continuous surveillance and monitoring efforts are essential for the timely detection and response to outbreaks, thereby enhancing the overall management and control of brucellosis in animal populations.

## 5. Conclusions

In conclusion, our study emphasizes the complex epidemiology of brucellosis in meat goat herds and highlights the importance of comprehensive disease management and control strategies. An integrated approach that includes proactive herd management practices, rigorous biosecurity measures, targeted educational campaigns, and collaborative research efforts is essential for reducing disease transmission, safeguarding herd health, and ensuring the sustainability of goat farming enterprises. For effective monitoring and surveillance programs targeting brucellosis, a combination of serological methods (such as mRBT and CFT) along with molecular methods is recommended to facilitate control and eradication efforts in endemic areas.

## Figures and Tables

**Table 1:** Intraherd prevalence of brucellosis in meat goat herds, Northeast Thailand.

No.	Seroprevalence % (numbers of seropositive/total sample)	Molecular prevalence % (numbers of PCR-positive/total sample)	Intraherd prevalence % (numbers of positive animal∗/total animal)
1	0.0 (0/28)	0.0 (0/4)	0.0 (0/28)
2	0.0 (0/22)	0.0 (0/5)	0.0 (0/22)
3	0.0 (0/16)	0.0 (0/7)	0.0 (0/16)
4	0.0 (0/12)	0.0 (0/1)	0.0 (0/12)
5	0.0 (0/20)	0.0 (0/2)	0.0 (0/20)
6	0.0 (0/13)	0.0 (0/7)	0.0 (0/13)
7	0.0 (0/13)	0.0 (0/6)	0.0 (0/13)
8	0.0 (0/6)	0.0 (0/5)	0.0 (0/6)
9	0.0 (0/8)	0.0 (0/1)	0.0 (0/8)
10	0.0 (0/10)	0.0 (0/4)	0.0 (0/10)
11	0.0 (0/2)	0.0 (0/1)	0.0 (0/2)
12	0.0 (0/9)	0.0 (0/7)	0.0 (0/9)
13	0.0 (0/7)	0.0 (0/7)	0.0 (0/7)
14	0.0 (0/8)	0.0 (0/7)	0.0 (0/8)
15	0.0 (0/11)	0.0 (0/10)	0.0 (0/11)
16	0.0 (0/6)	0.0 (0/4)	0.0 (0/6)
17	0.0 (0/6)	0.0 (0/5)	0.0 (0/6)
18	0.0 (0/7)	0.0 (0/7)	0.0 (0/7)
19	0.0 (0/4)	0.0 (0/2)	0.0 (0/4)
20	0.0 (0/16)	0.0 (0/15)	0.0 (0/16)
21	0.0 (0/13)	0.0 (0/12)	0.0 (0/13)
22	0.0 (0/7)	0.0 (0/6)	0.0 (0/7)
23	0.0 (0/9)	0.0 (0/8)	0.0 (0/9)
24	0.0 (0/8)	0.0 (0/7)	0.0 (0/8)
25	0.0 (0/4)	0.0 (0/1)	0.0 (0/4)
26	0.0 (0/10)	0.0 (0/9)	0.0 (0/10)
27	0.0 (0/17)	0.0 (0/17)	0.0 (0/17)
28	0.0 (0/9)	0.0 (0/9)	0.0 (0/9)
29	0.0 (0/13)	0.0 (0/12)	0.0 (0/13)
30	0.0 (0/22)	0.0 (0/20)	0.0 (0/22)
31	0.0 (0/9)	Not done	0.0 (0/9)
32	0.0 (0/32)	10.0 (1/10)	3.13 (1/32)
33	0.0 (0/24)	4.35 (1/23)	4.16 (1/24)
34	0.0 (0/20)	20.0 (1/5)	5.00 (1/20)
35	0.0 (0/14)	7.69 (1/13)	7.14 (1/14)
36	0.0 (0/14)	8.33 (1/12)	7.14 (1/14)
37	0.0 (0/11)	10.00 (1/10)	9.09 (1/11)
38	0.0 (0/44)	10.26 (4/39)	9.09 (4/44)
39	0.0 (0/11)	14.29 (1/7)	9.09 (1/11)
Total	0.0 (0/515)	0–20.0 (11/327)	0–9.09 (11/515)

^∗^Positive animal = seropositivity or positive PCR assay within an animal.

**Table 2:** Univariate analysis examining herd-level risk factors associated with the presence of brucellosis in meat goat herds in Northeast Thailand.

Risk factor	Category	Total (%)	Prev. (%)	OR	95% CI	*p* value
• Herd structure						
Herd established (years)	< 5	20 (51.3)	3/39 (7.7)			
	> 5	19 (48.7)	5/39 (12.8)	2.02	0.41–9.99	0.380

Herd size	< 12	23 (59.0)	2/39 (5.1)			
	> 13	16 (41.0)	6/39 (15.4)	**6.30**	**1.07–36.93**	**0.041**

• Herd and health management practices						
Pasture	Only meat goat	21 (53.8)	4/39 (10.3)			
	Mixed with other species	18 (46.2)	4/39 (10.3)	1.21	0.25–5.75	0.806

Presence of other ruminant livestock	No	20 (51.3)	5/39 (12.8)			
	Yes	19 (48.7)	3/39 (7.7)	0.56	0.11–2.77	0.479

Good farm management practice	Approved	11 (28.2)	3/39 (7.7)			
	Unapproved	28 (71.8)	5/39 (12.8)	0.58	0.11–2.99	0.515

Brucellosis testing annually in herd (serology test)	Yes	13 (33.3)	3/39 (7.7)			
	No	26 (66.7)	5/39 (12.8)	0.79	0.15–3.99	0.779

Brucellosis testing before movement	Yes	7 (17.9)	2/39 (5.1)			
	No	32 (82.1)	6/39 (15.4)	0.58	0.08–3.72	0.563

Introduced a new animal in herds within 6 months	Yes	25 (64.1)	6/39 (15.4)	1.89	0.32–10.96	0.475
	No	14 (35.9)	2/39 (5.1)			

Quarantine a new animal for at least 30 days	Yes	5 (12.8)	1/39 (2.6)			
	No	34 (87.2)	7/39 (17.9)	1.04	0.99–10.80	0.975

Buck circulation between herds	Not used	13 (33.3)	3/39 (7.7)			
	Used	26 (66.7)	5/39 (12.8)	0.40	0.07–2.15	0.286

Duration of buck in a farm (years)	< 3	29 (74.4)	6/39 (15.4)			
	> 3	9 (23.1)	2/39 (5.1)	0.95	0.15–5.74	0.962

Culling of used buck	To slaughterhouse	9 (23.1)	2/39 (5.1)			
	To another farm to use	30 (76.9)	6/39 (15.4)	0.88	0.14–5.33	0.884

Separate parturition area	Yes	11 (28.2)	3/39 (7.7)			
	No	28 (71.8)	5/39 (12.8)	0.68	0.13–3.47	0.644

Mother-rearing system	Yes	39 (100.0)	8/39 (20.5)	0.27	0.005–14.62	0.520
	No	0 (0.0)	0/39 (0.0)			

Wear gloves during assistance doe when giving birth	Yes	11 (28.2)	1/39 (2.6)			
	No	28 (71.8)	7/39 (17.9)	3.33	0.35–30.89	0.289

Wear a mask during assistance doe when giving birth	Yes	2 (5.1)	1/39 (2.6)			
	No	37 (94.9)	7/39 (17.9)	0.23	0.01–4.20	0.323

Presence of rodent	Yes	27 (69.2)	8/39 (20.5)	10.90	0.57–205.99	0.111
	No	12 (30.8)	0/39 (0.0)			

Presence of dogs or cats	Yes	32 (82.1)	6/39 (15.4)	0.58	0.08–3.72	0.563
	No	7 (17.9)	2/39 (5.1)			

Manure uses	Fertilizer	35 (89.7)	6/39 (15.4)	0.20	0.02–1.77	0.150
	Not used	4 (10.3)	2/39 (5.1)			

• Reproductive history						
Abortion history in the herd	No	17 (43.6)	1/39 (2.6)			
	Yes	22 (56.4)	7/39 (17.9)	7.46	0.81–68.10	0.074

Repeat breeding history in the herd	No	34 (87.2)	7/39 (17.9)			
	Yes	5 (12.8)	1/39 (2.6)	0.96	0.09–10.04	0.975

Sterile history in the herd	No	28 (71.8)	5/39 (12.8)			
	Yes	11 (28.2)	3/39 (7.7)	1.72	0.33–8.91	0.515

Orchitis history in the herd	No	35 (89.7)	7/39 (17.9)			
	Yes	4 (10.3)	1/39 (2.6)	1.33	0.11–14.84	0.815

Weak kids' history in the herd	No	17 (43.6)	3/39 (7.7)			
	Yes	20 (56.4)	5/39 (12.8)	1.37	0.27–6.77	0.697

Abortion or repeat breeding or sterile with weak kids' history in the herd	3-4 occasional	5 (12.8)	3/34 (8.82)	**9.37**	**1.17–74.84**	**0.034**
	1-2 occasional	29 (74.4)	4/34 (11.76)			

• Knowledge of transmission modes and history of clinical signs associated with brucellosis in humans						
Signs of fever, sweats, or muscle aches like brucellosis in human	No	33 (84.6)	7/39 (17.9)			
	Yes	6 (15.4)	1/39 (2.6)	0.74	0.07–7.43	0.800

Knowledge of direct contact transmission	Not know	23 (59.0)	1/39 (2.6)			
	Know	16 (41.0)	7/39 (17.9)	**0.05**	**0.006–0.54**	**0.012**

Knowledge of ingestion transmission	Not know	36 (92.3)	7/39 (17.9)			
	Know	3 (7.7)	1/39 (2.6)	0.48	0.03–6.11	0.570

Knowledge of inhalation transmission	Not know	37 (94.9)	7/39 (17.9)			
	Know	2 (5.1)	1/39 (2.6)	0.23	0.01–4.20	0.323

*Note:* Prevalence (Prev.), odds ratio (OR), and 95% confidence interval (95% CI) of the variable from 8 positive herds (20.51%) based on a total sample from 39 herds. Bold values indicate a significant association, classified as *p* < 0.05.

**Table 3:** Multivariable analysis of herd-level risk factors associated with brucellosis among meat goat herds in Northeast Thailand.

Risk factor	Category	OR	95% CI	*p* value
Herd size	< 12			
	> 13	**10.20**	**1.07–97.40**	**0.044**

*Note:* Factors including abortion or repeat breeding or sterile together with weak kids' history in the herd were excluded from the final multivariable regression model (*p* > 0.05). Bold values indicate a significant association, classified as *p* < 0.05.

**Table 4:** Univariate analysis of individual-level risk factors associated with brucellosis in meat goat populations in Northeast Thailand.

Risk factor	Category	Total (%)	Prev. (%)	OR	95% CI	*p* value
Gender	Female	475 (92.2)	11/515 (2.1)	2.00	0.11–34.65	0.632
	Male	40 (7.8)	0/515 (0.0)			

Age	> 1 year	491 (95.3)	11/515 (2.1)	1.17	0.07–20.48	0.913
	< 1 year	24 (4.7)	0/515 (0.0)			

BCS	Thin—emaciated	308 (59.8)	8/515 (1.6)	1.81	0.47–6.91	0.383
	Average—fat	207 (40.2)	3/515 (0.6)			

*Note:* Prevalence (Prev.), odds ratio (OR), and 95% confidence interval (95%CI) of the variable from a total sample of 515 animals; positive PCR assay 11/327 (3.36%).

## Data Availability

The data that support the findings of this study are available from the corresponding author upon reasonable request.

## Supporting Information

Supporting Information Supporting 1: Questionnaires used for interviewing goat farmers.

Supporting Information Figure S1: Sensitivity of the real-time PCR assay, Figure S2: Real-time PCR shows samples positive for *Brucella* spp., Figure S3: Nucleotide sequence and Chromatogram of PCR product of the selected positive sample, Figure S4: Blast search results and Distance tree of the nucleotide sequence of the positive sample.
