# Supplementary material for: Serological and Molecular Prevalence and Associated Risk Factors in Caprine Brucellosis, Northeastern Thailand
Source: Vet Med Int. 2024 Oct 23;2024:9966352. doi: 10.1155/2024/9966352 (PMC11524711; doi:10.1155/2024/9966352)
Supplement: Supporting Information — Supporting 1: Questionnaires used for interviewing goat farmers. [file 9966352.f1.docx]

**Questionnaires (Brucellosis)**

**Herd structure**

1. How many years have you been raising goats? ……. years.
2. How many mature animals are in the herd (aged > 6 months) ? Total:………. Animals

**Herd and health management practices**

1. Pasture Characteristics:

□ The pasture is also utilized by other animal species, such as cattle, goats, sheep, or buffalo.

□ The pasture is exclusively used by the specified species, with no other animals present.

1. In your farm, do you currently raise other livestock? (You may select more than one option)

□ Beef cattle □ Buffalo □ Sheep □Pigs □Chicken □None / Do not raise other animals

1. Do you have an approved certification for Good Farm Management Practice from the Department of Livestock Development?

□ Yes, approved

□ No, unapproved

1. Do you conduct annual Brucellosis testing (serology test) for your herd?

□ Yes

□ No

1. Do you perform Brucellosis testing before the movement of animals?

□ Yes

□ No

1. Have you introduced any new animals into the herd within the past 6 months?

□ Yes

□ No

1. Do you quarantine new animals for at least 30 days?

□ Yes

□ No

1. Breeding Male Utilization on the Farm (You may select more than one option)

□ Used exclusively on the farm

□ Circulate within a communal of farms / Occasionally borrow breeding males from other farms

1. How many years do you use a breeding male on your farm before culling him?

□ < 1 year

□ 1-2 years

□ > 3 years

1. What is done with breeding males that are no longer in use?

□ Selling to a slaughterhouse

□ Selling to another farm

1. Do you have a separate area for parturition?

□ Yes

□ No

1. How do you handle newborn goat kids until weaning?

□ mother rearing system

□ non-mother rearing system

1. Do you wear gloves when assisting a doe during giving birth?

□ Yes

□ No

1. Do you wear a mask during assistance a doe when giving birth?

□ Yes

□ No

1. Is there a presence of rodents on the farm?

□ Yes

□ No

1. Is there a presence of dogs on the farm?

□ Yes

□ No

1. Is there a presence of cats on the farm?

□ Yes

□ No

1. How is manure used?

□ For fertilizer

□ Not use

**Reproductive history**

1. Have you ever observed cases of abortion in the herd?

□ Yes

□ No

1. Have you ever observed cases of repeat breeding in the herd?

□ Yes

□ No

1. Have you ever observed cases of sterility in the herd?

□ Yes

□ No

1. Have you ever observed cases of orchitis in the herd?

□ Yes

□ No

1. Have you ever observed cases of weak born kids in the herd?

□ Yes

□ No

**Knowledge of transmission modes and history of clinical signs associated with brucellosis in human**

1. Have you ever exhibited symptoms such as fever, sweating, or muscle aches that resemble those of brucellosis in humans?

□ Yes

□ No

1. Do you know that brucellosis can be transmitted to humans via direct contact?

□ know

□ not know

1. Do you know that brucellosis can be transmitted to humans via ingestion?

□ know

□ not know

1. Do you know that brucellosis can be transmitted to humans via inhalation?

□ know

□ not know
