# Supplementary material for: Serological and Molecular Prevalence and Associated Risk Factors in Caprine Brucellosis, Northeastern Thailand
Source: Vet Med Int. 2024 Oct 23;2024:9966352. doi: 10.1155/2024/9966352 (PMC11524711; doi:10.1155/2024/9966352)
Supplement: Supporting Information — Figure S1: Sensitivity of the real-time PCR assay, Figure S2: Real-time PCR shows samples positive for Brucella spp., Figure S3: Nucleotide sequence and Chromatogram of PCR product of the selected positive sample, Figure S4: Blast search results and Distance tree of the nucleotide sequence of the positive sample. [file 9966352.f2.docx]

**Supplementary figures**

**
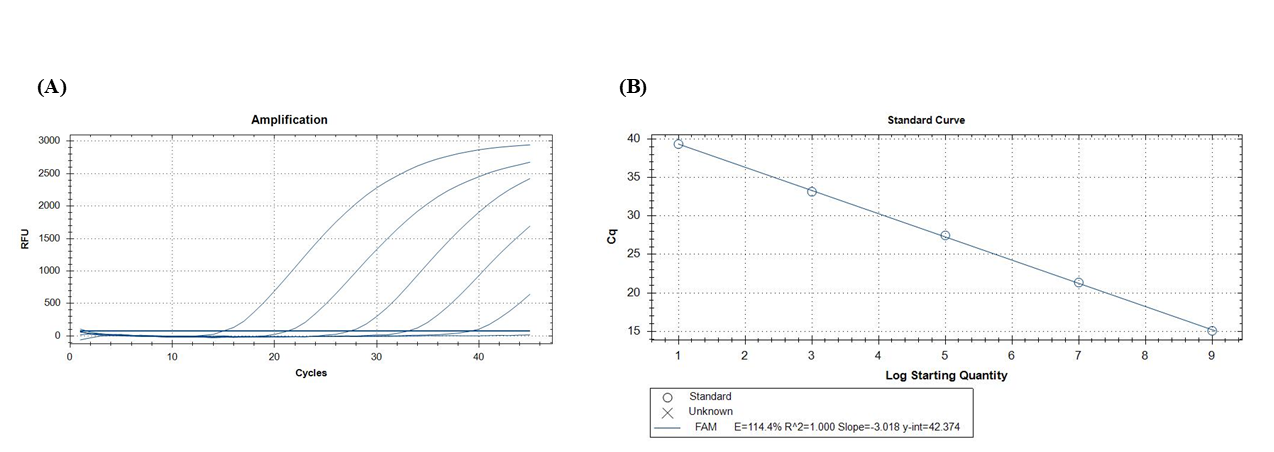
**

**Figure S1.** Sensitivity of the real-time PCR assay. (A) Amplification plots of the real-time PCR assay performed on diluted plasmid DNA at copy numbers of 10^9^, 10^7^, 10^5^, 10^3^ and 10. (B) Standard curve derived by plotting the threshold cycle (C_t_) values against the copy number of plasmid DNA.


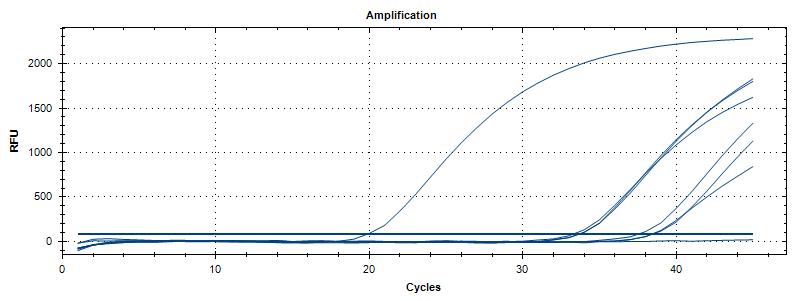


**Figure S2**. Real-time PCR shows samples positive for *Brucella* spp.

>356-BSCPP-F

CGGCTTTACGCAGTCAGACGTTGCCTATTGGGCCTATAACGGCACCGGCCTTTATGATGGCAAGGGCAAGGTGGAAGATTTGCGCCTTCTGGCGACGCTTTACC


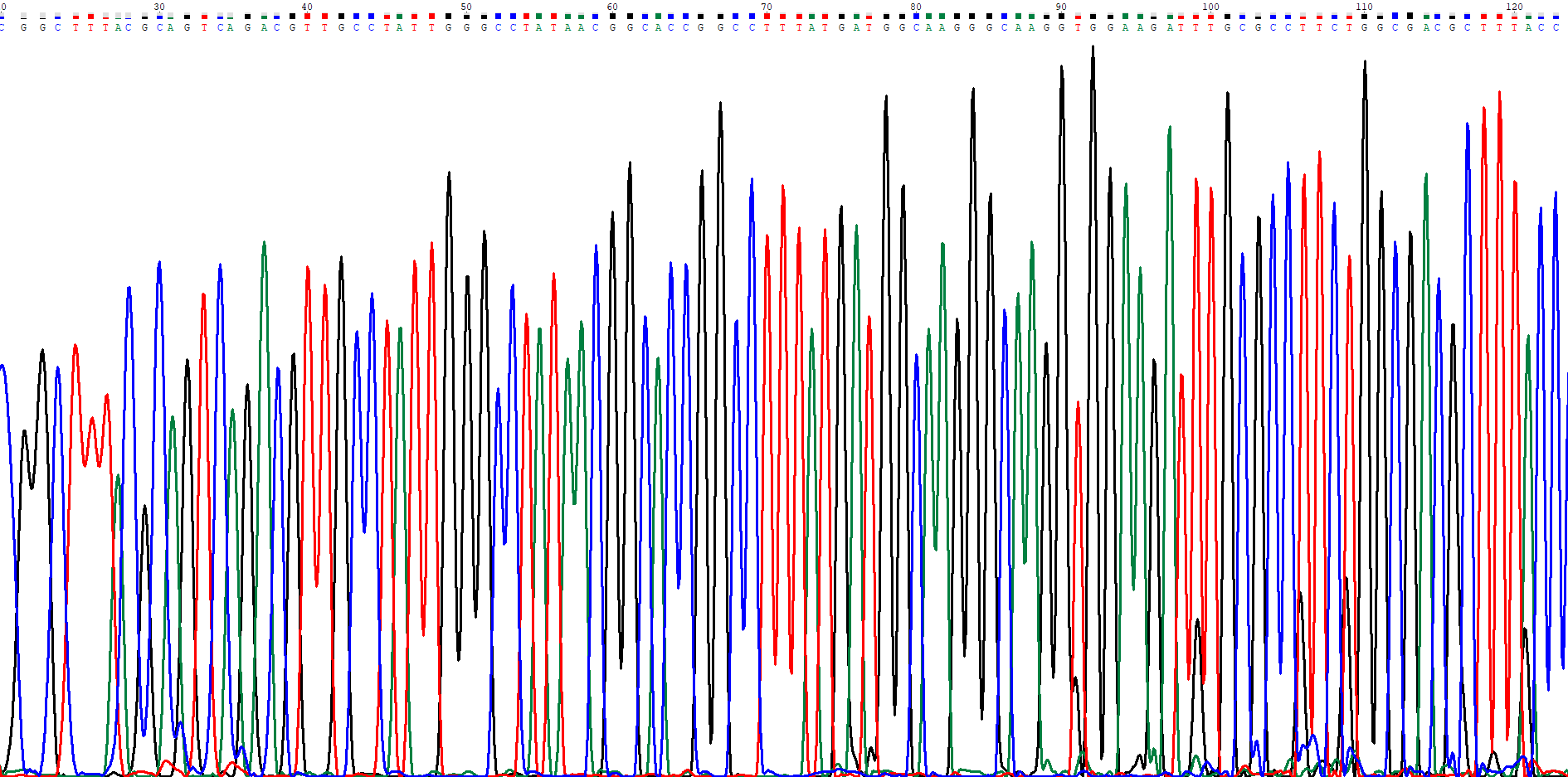


**Figure S3.** Nucleotide sequence and Chromatogram of PCR product of the selected positive sample.


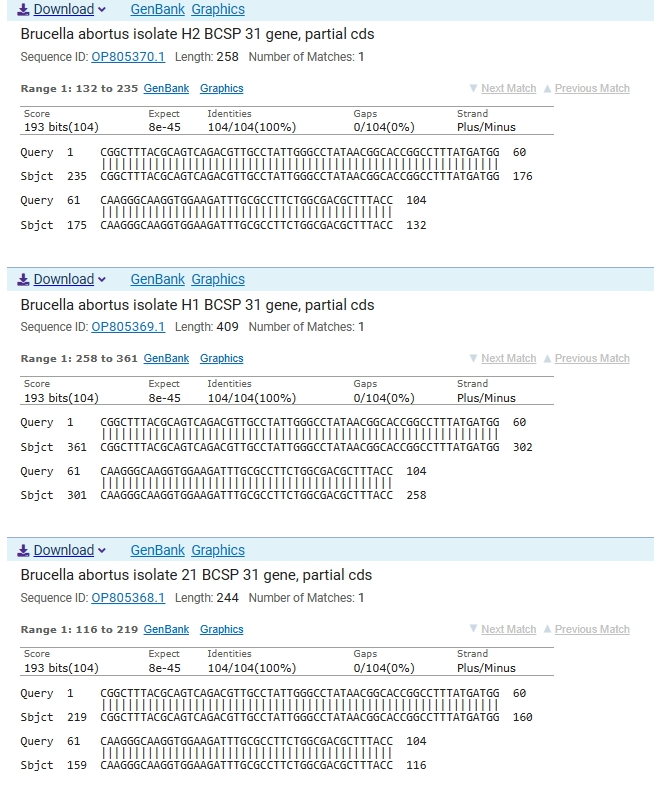


356


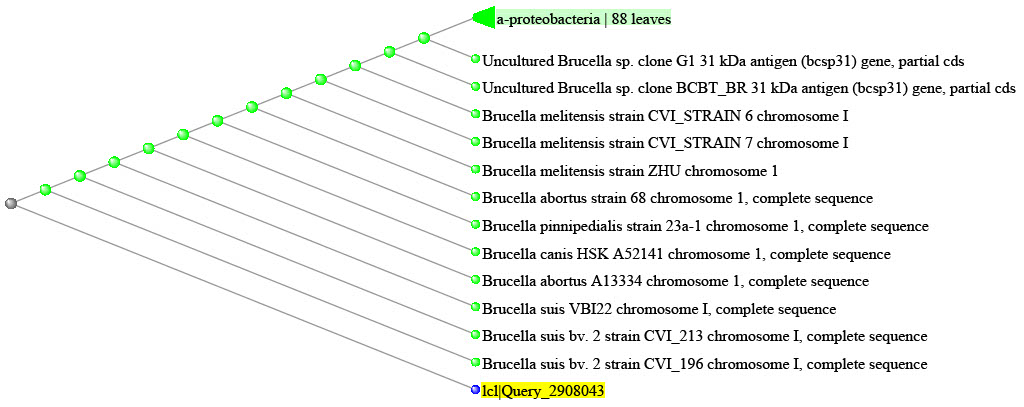


**Figure S4.** Blast search results and Distance tree of the nucleotide sequence of the positive sample.
